# Reconstitution of the myeloid and lymphoid compartments after the transplantation of autologous and genetically modified CD34^+ ^bone marrow cells, following gamma irradiation in cynomolgus macaques

**DOI:** 10.1186/1742-4690-5-50

**Published:** 2008-06-19

**Authors:** Sonia Derdouch, Wilfried Gay, Didier Nègre, Stéphane Prost, Mikael Le Dantec, Benoît Delache, Gwenaelle Auregan, Thibault Andrieu, Jean-Jacques Leplat, François-Loïc Cosset, Roger Le Grand

**Affiliations:** 1 CEA, service d'Immuno-Virologie, Institut des Maladies Emergentes et Thérapies Innovantes, Direction des Sciences du Vivant, Fontenay aux Roses, France; 2 Université Paris-Sud, UMR-E01, Orsay, France; 3 Université de Lyon, (UCB-Lyon1), IFR128, Lyon, F-69007, France; 4 INSERM, U758, Lyon, F-69007, France; 5 Ecole Normale Supérieure de Lyon, Lyon, F-69007, France; 6 CEA, DSV, IRCM, SREIT, Laboratoire de Radiobiologie et d'Etude du Génome, Jouy-en-Josas, F-78352 France; 7 INRA, DGA, Laboratoire de Radiobiologie et d'Etude du Génome, Jouy-en-Josas, F-78352 France

## Abstract

**Background:**

Prolonged, altered hematopoietic reconstitution is commonly observed in patients undergoing myeloablative conditioning and bone marrow and/or mobilized peripheral blood-derived stem cell transplantation. We studied the reconstitution of myeloid and lymphoid compartments after the transplantation of autologous CD34^+ ^bone marrow cells following gamma irradiation in cynomolgus macaques.

**Results:**

The bone marrow cells were first transduced *ex vivo *with a lentiviral vector encoding eGFP, with a mean efficiency of 72% ± 4%. The vector used was derived from the simian immunodeficiency lentivirus SIVmac251, VSV-g pseudotyped and encoded eGFP under the control of the phosphoglycerate kinase promoter. After myeloid differentiation, GFP was detected in colony-forming cells (37% ± 10%). A previous study showed that transduction rates did not differ significantly between colony-forming cells and immature cells capable of initiating long-term cultures, indicating that progenitor cells and highly immature hematopoietic cells were transduced with similar efficiency. Blood cells producingeGFP were detected as early as three days after transplantation, and eGFP-producing granulocyte and mononuclear cells persisted for more than one year in the periphery.

**Conclusion:**

The transplantation of CD34^+ ^bone marrow cells had beneficial effects for the *ex vivo *proliferation and differentiation of hematopoietic progenitors, favoring reconstitution of the T- and B-lymphocyte, thrombocyte and red blood cell compartments.

## Background

Gene therapy strategies hold promise for the treatment of hematopoietic disorders. All hematopoietic lineages, including polymorphonuclear cells, monocytes, lymphocytes and natural killer cells, and hematopoietic stem cells (HSC) – which are capable of self-renewal and pluripotent differentiation – have been targeted for transduction with therapeutic genes. Most diseases for which gene therapy could be proposed require stable and long-lasting transgene expression for efficacy. Retroviral vectors present the major advantage of integrating the transferred DNA stably into the genome of target cells, which is then passed on to progeny. However, they cannot infect and integrate into non dividing cells[1]. Most HSC are quiescent [2], respond slowly to stimulation [3-7] and tend to differentiate and lose their repopulating capacity upon stimulation[3,8-11]. Lentiviral vectors can be used to transduce cells in growth arrest [12]*in vivo *and *ex vivo*[13], thanks to interaction of the preintegration complex – composed of viral VPX and integrase proteins – with the nuclear pore complex[14]. Vectors derived from HIV-1[15,16], HIV-2[17], FIV[18] and equine infectious anemia virus (EIAV)have been tested[19].

Methods for transferring genes into hematopoietic cells must be tested in relevant animal models before their application to humans [20,21]. Studies in nonhuman primates (NH)P provide an ideal compromise, because these species are phylogenetically closely related to humans and a high level of nucleotide sequence identity is observed between the genes encoding many hematopoietic growth factors and cytokines in these mammals and their counterparts in humans[22]. Moreover, hematopoiesis in macaques is very similar to that in humans, and the HSC biology of macaques is much more similar to that of humans than is that of rodents, making macaques good candidates for hematopoietic stem cell engraftment studies [23-26]. In addition, testing lentiviral based gene transfer strategies need to be assessed in species that are susceptible to lentivirus induced disease. Or particular interest are the Feline immunodeficiency virus (FIV) infection which causes a clinical disease in cats that is remarkably similar to HIV disease in human [27-30] and experimental infection of macaques with the simian immunodeficiency virus (SIV) reproducing both chronic infection and an AIDS-like disease very similar to those observed in human patients infected with HIV. Despite the theoretical advantages of lentiviral vectors over oncoretroviral vectors, non human primate lentiviruses clearly have pathogenic properties [31]. The use of lentiviral vectors derived from potentially pathogenic primate lentiviruses, such as SIV, therefore continues to raise serious clinical acceptance concerns. SIV-based vectors, such as SIVmac239[31,32] and SIVmac251[33,34], may provide a unique opportunity to test the safety and efficacy of primate lentiviral vectors *in vivo*.

Recent improvements in the efficiency of gene transfer to NHP repopulating cells[11,35,36] have provided new opportunities to follow the progeny of each primitive progenitor and stem cells directly *in vivo*, using retroviral marking to track individual progenitor or stem cell clones[37]. Clinically relevant levels (around 10%) of genetically modified cells in the peripheral blood have been achieved by *ex vivo *gene transfer into HSC and the autologous transplantation of these cells into macaques[37]. Successful and persistent engraftment (up to six months) has also been reported in non human primates with primitive CD34^+ ^progenitors genetically modified with a murine retrovirus vector encoding the murine CD24 gene as a reporter gene[38]. In both trials, marked cells of multiple hematopoietic lineages were identified in the blood: granulocytes, monocytes and B and T cells, including naive T lymphocytes[37,38]. The efficacy of HSC gene transfer could theoretically be improved by the use of newly developed retroviral or lentiviral vectors. Particles bearing an alternative envelope protein, such as that of the feline endogenous virus (RD114), have been shown to be superior to amphotropic vectors for the transduction of NHP stem cells followed by autologous transplantation [39,40].

We report here the results obtained *in vitro *and *in vivo *in an experiment assessing the efficacy and safety of a gene transfer protocol based on the transduction of simian CD34^+ ^bone marrow cells with a minimal SIVmac251-derived lentiviral vector. This system is based on the VSVg-pseudotyped SIV vector encoding enhanced green fluorescent protein (eGFP) under control of the phosphoglycerate kinase (PGK) promoter. Most immature CD34^+ ^hematopoietic cells capable of initiating long-term culture (LTC-IC) were efficiently transduced, and eGFP-positive cells were detectable *in vivo *in all animals more than one year after transplantation.

## Methods

### Animals

Male cynomolgus macaques (*Macaca fascicularis*), weighing between 3 and 6 kg were imported from Mauritius and housed in single cages within level 3 biosafety facilities, according to national institutional guidelines (*Commission de génie génétique*, Paris, France). All experimental procedures were carried out in accordance with European guidelines for primate experiments (*Journal Officiel des Communautés Européennes*, L358, December 18 1986).

### Immunoselection of non human primate CD34^+ ^bone marrow progenitor cells

Bone marrow mononuclear cells were obtained from the iliac crest or by aspiration from the humerus and isolated by standard Ficoll density-gradient centrifugation (MSL2000, Eurobio, Les Ulis, France). Cells were washed twice in phosphate-buffered saline (PBS, Eurobio, Les Ulis, France) and resuspended in 1% FCS (Fetal Calf Serum; Bio West, France) in PBS. The cellular fraction was then enriched in CD34^+ ^cells by positive immunomagnetic selection, using beads coupled to a specific antibody (clone 561; Dynabeads M-450 CD34, Progenitor Cell Selection System, Dynal, Oslo, Norway), according to the manufacturer' s instructions. Immunoselected CD34^+ ^cells were stained with a specific PE-conjugated anti-CD34 antibody (clone 563; Pharmingen, Becton Dickinson, California, USA) and analyzed by flow cytometry (LSR, Becton Dickinson, California, USA) to evaluate the level of enrichment. All preparations contained more than95% CD34^+ ^cells, with a mean value of 97% ± 1% (n = 12) for *in vitro *assays and 96% ± 1% (n = 4) for *in vivo *assay.

### Lentiviral vector

**Figure 1:**
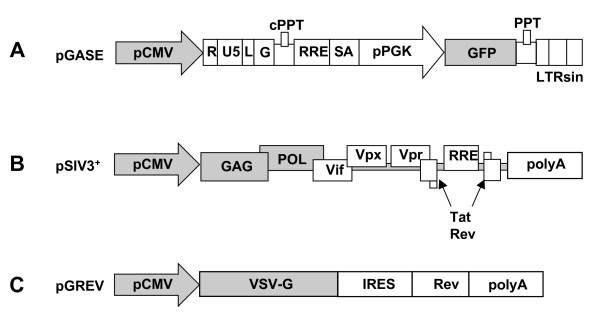
Schematic representation of SIV-derived SIN vector, helper construct and VSV-g encoding plasmid. An SIVmac251-derived vector was produced by cotransfecting 293T cells with three plasmids: A. a plasmid pGASE containing the eGFP gene under control of the PGK promoter; B. a plasmid pSIV3+ containing viral genes; C. a plasmid pGREV containing the VSV envelope gene. Cis genetic elements are symbolized with white boxes, whereas promoters and genes are depicted by shadowed boxes. pCMV, early cytomegalovirus promoter; pPGK, mouse phosphoglycerate kinase-1 promoter; RRE, REV-responsible element; SA, SIV Rev/Tat splice acceptor; cPPT and PPT, central and 3' polypurine tracks, respectively; GFP, the gene encoding the enhanced green fluorescent protein; LTRsin, partially U3 deleted 3'LTR; LG, leader and a 5' GAG region.

Two SIV-derived vectors were produced, one for *in vitro *studies and the other for *in vivo *studies: 1) pRMES8 is a minimal packaging-competent SIVmac251-based vector[34]. It contains the enhanced green fluorescent protein (eGFP) marker gene under control of the mouse phosphoglycerate kinase (PGK) promoter, placed between the SIVmac251 LTRs and leader sequences. It carries the SIVmac251 RRE region and minimal sequences of the *gag *and *pol *genes encompassing central polypurine tract/central termination sequence (cPPT/CTS) regions (figure 1A). pRMES8 was used for *in vitro *assays investigating the susceptibility of CD34^+ ^cells from primate bone marrow to transduction with SIVmac251-derived vectors. 2) For *in vivo *assays, we used pGASE; this plasmid is an optimised version of pRMES8, with a 3'-SIN-LTR for safety and insertion of an exon splicing enhancer (ESE) upstream the PGK promoter to increase titer [41]

pSIV3^+ ^is the packaging plasmid derived from the BK28 molecular clone of SIVmac251, as described elsewhere[33]. Briefly, the pSIV3^+ ^*gag*/*pol *expression plasmid was obtained by replacing the 5' LTR of SIVmac251 (nucletotides 1 to 506) by the human cytomegalovirus (CMV) early-immediate promoter and enhancer region. The 5' half of the *env *gene (nt 6582 to 7981) was also removed, leaving the RRE (REV-responsive element) sequence and the 5' and 3' exons of the *tat *and *rev *regulatory genes intact. The 3' LTR (nt 9444 to 10249) was replaced by a SV40 polyadenylation sequence, resulting in deletion of the 3' end of the *nef *gene. Finally, the *nef *initiation codon was inactivated to prevent translation (figure 1B).

pGREV was used for pseudotyping. It is a bicistronic expression construct encoding the vesicular stomatitis virus glycoprotein (VSV-g) and the REV regulatory protein, linked by an EMCV IRES. Expression of this cassette, which contains the rabbit β-globin intron II and polyadenylation (pA) sequences (figure 1C), is driven by the constitutive CMV promoter.

### Production of SIV vectors

293T cells were plated at a density of 4.0 × 10^5 ^cells per well (in 6-well plates) on the day before transfection. Cells were transfected as previously described[42]. SIV vectors were produced by cotransfection with three plasmids: the SIV plasmid vector (pRMES8 or pGASE)(1.7 μg), the helper plasmid, pSIV3^+^, encoding Gag-Pol and regulatory proteins other than Env and Nef (1.7 μg) and the envelope glycoprotein-encoding plasmid pGREV (2.2 μg). The transfection medium was replaced after 16 hours of incubation. Virus-containing medium was collected 40 hours after transfection, clarified by centrifugation for 5 minutes at 800 g, and passed through a filter with 0.45 μm pores. For high-titer preparations, SIV vectors were concentrated by ultracentrifugation at 110,000 g for 2 hours. The viral pellet was resuspended by incubation for 2 hours at 4°C in phosphate-buffered saline supplemented with 1% glycerol[34].

For determination of the infectious titer, sMAGI cells were seeded at a density of 4 × 10^5 ^cells/ml in six-well plates one day before transduction in DMEM medium (Life Technologies Inc., Berlin, Germany) supplemented with 10% fetal bovine serum (FBS) (Gibco BRL, Grand Island, New York, USA), polybrene (6 μg/ml) (Sigma, Saint Louis, USA) and an antibiotic mixture (5 mg/ml penicillin; 5 mg/ml streptomycin; 10 mg/ml neomycin; Gibco BRL, Grand Island, New York, USA). The cells were cultured for one day, and we then added serial dilutions of virus preparations and incubated the plates for a further four hours. Cells were then washed in DMEM (Life Technologies Inc., Berlin, Germany). Transduction rates was determined 48 hours after infection, as the percentage of GFP-positive sMAGI cells (%GFP^+^c), by flow cytometry (FACScan, Becton Dickinson, San Jose, Mountain View, California, USA) after transducing 4 × 10^5 ^cells with 1 ml of diluted viral supernatant (dilution factor = d). The infectious titer (IT), expressed as transducing units/ml, was calculated as: IT = %GFP^+^cells × 4 × 10^5^/100 × d.

### Transduction of immunoselected CD34^+ ^cells

Following immunoselection, CD34^+ ^cells were cultured in a proliferation medium composed of Iscove's MDM supplemented with 1% bovine serum albumin (BSA), bovine pancreatic insulin (10 μg/ml), human transferrin (200 μg/ml), 2-mercaptoethanol (10^-4^M) and L-glutamine (2 mM; Stemspan, Stem Cell Technologies, Meylan, France). The medium was supplemented with 50 ng/ml recombinant human (rh) SCF (Stem Cell Technologies, Meylan, France), 50 ng/ml rh Flt3-L (Stem Cell Technologies, Meylan, France), 10 ng/ml rh IL-3 (R&D Systems, Minneapolis, USA),10 ng/ml rh IL-6 (R&D Systems, Minneapolis, USA) and 4 μg/ml polybrene (Sigma, Saint Louis, USA) in plates coated with retronectin (Cambrex Bio Science, Paris, France). The CD34^+^cells were then transduced by 24 hours of coculture with the vector (multiplicity of infection (MOI) = 100).

### Myeloid differentiation of CD34^+ ^cells

Following the coculture of CD34^+ ^cells with lentiviral vector, part of the cell culture was fixed in CellFix solution (Becton Dickinson, Erembodegem, Belgium) for evaluation of the rate of transduction of undifferentiated CD34^+ ^cells. Part of the cell culture was cultured for 14 days in 35 mm Petri dishes containing semi-solid medium (Methocult GF H4434, Stem Cell Technologies, Meylan, France) composed of Iscove's MDM medium supplemented with 1% methylcellulose, 30% fetal bovine serum, 10^-4 ^M 2-mercaptoethanol, 2 mM L-glutamine, 50 ng/ml rhSCF, 10 ng/ml rhGM-CSF, 10 ng/ml rhIL-3 and 3 IU/ml rhEPO. Cells were cultured at a density of 10^4 ^cells/ml (in triplicate) at 37°C, under an atmosphere containing 5% CO_2_, to allow the myeloid differentiation of colony-forming cells (CFC).

The remaining cells were cocultured in 96-well plates for 35 days at 37°C, under an atmosphere containing 5% CO_2_, on a layer of stromal cells of the murine fibroblastic cell line M2-10B4, in a medium composed of αMEM supplemented with 12.5% horse serum (HS), 12.5% FBS, 2 mM L-glutamine, 10^-4 ^M 2-mercaptoethanol, 0.16 M I-inositol and 16 μM folic acid (Myelocult H5100, Stem Cell Technologies, Meylan, France) and 10^-6 ^M hydrocortisone. Cells were cultured at a concentration of 10^3 ^cells per well (24 wells per condition per monkey), to allow long-term culture-initiating cells (LTC-IC) to undergo myeloid differentiation to generate progenitor cells or CFC. The CFC were cultured for 14 days on semi-solid medium, as described above, to allow their myeloid differentiation into more mature cells.

### AZT pretreatment of immunoselected CD34^+ ^cells

CD34^+ ^cells were treated with AZT before transduction, to inhibit transduction due to reverse transcription of the lentiviral vector genome. Immunoselected CD34^+ ^cells were cultured overnight in the proliferation medium described above, with AZT concentrations of 0, 10^-7^, 10^-6 ^and 10^-5 ^molar. The cells were washed twice and transduced with the lentiviral vector, according to the protocol described above. The real percentage of GFP-positive cells resulting from reverse transcription of the lentiviral vector was thus determined by subtracting the percentage of GFP-positive cells obtained after treatment with a saturating dose of AZT, from the percentage of GFP-positive cells obtained in the absence of AZT treatment.

### Fluorescence microscopy

After transduction and myeloid differentiation in semi-solid medium, the colonies formed by AZT-treated CFC were observed by fluorescence microscopy (Axiovert S100, Zeiss) using a magnification factor of 100. Fluorescence microscopy was used to detect GFP in each colony subtype, making it possible to determine the percentage of the colonies positive for GFP. We considered all colonies containing GFP-producing cells to be GFP-positive. Images were analyzed with Adobe Premiere and Adobe Photoshop software (Adobe Systems Inc., San Jose, CA, USA).

### Gamma irradiation

Eight animals were sedated with ketamine (Imalgène; 10 mg/kg, i.m.), Rhône-Mérieux, France) and placed in a restraint chair. They received myeloablative conditioning, in the form of total body exposure to ^60^Co gamma rays with an anterior unilateral direction. A total midline tissue dose of 6 Gy was delivered at a rate of 25.92 cGy/minute. Dosimetry was performed, with 100 μL ionization chambers placed in paraffin wax cylindrical phantoms of a similar size and orientation to the seated animal.

### Transplantation of modified CD34^+ ^bone marrow cells

After the coculture of CD34^+ ^cells with the lentiviral vector, four animals underwent intramedullary infusion, of whole immunoselected CD34^+ ^cells into both humeri (Table 1).

**Table 1:** Reconstitution with transduced autologous CD34^+ ^cells in irradiated cynomolgus macaques

Monkeys	CD34^+ ^cells purity	CD34^+ ^cells collected	CD34^+ ^cells transduced	CD34^+ ^cells infused/kg
6653	96.42%	8.8 × 10^6^	76.54%	2.96 × 10^6^
6833	95.85%	8.0 × 10^6^	67.74%	1.50 × 10^6^
6896	95.46%	7.3 × 10^6^	67.76%	1.47 × 10^6^
7036	97.08%	5.5 × 10^6^	74.22%	1.46 × 10^6^

### Clinical support

All animals received clinical support in the form of antibiotics and fresh irradiated whole blood, as required. An prophylactic antibiotic regimen was initiated when leukocyte count fell below 1,000/μl and continued daily until it exceeded 1,000/μl for three consecutive days: 1 ml/10 kg/day Bi-Gental^® ^(Schering-Plough Santé Animale) and 1 ml/10 kg Terramycin^® ^(Pfizer). Fresh, irradiated (25 Gy; ^137^Cs gamma radiation) whole blood (approximately 50 ml/transfusion) from a random donor pool was administered if platelet count fell below 20,000/μl and hemoglobin concentration was less than 6 g/dl.

### Flow cytometry analysis

Peripheral blood and bone marrow mononuclear cells were incubated for 30 min at 4°C with 10 μl of selected monoclonal antibodies for single- or triple-color membrane staining. The following antibodies were used: APC-conjugated anti-CD3 (SP34-2, Becton Dickinson), PE-conjugated anti-CD14 (clone M5E2, BD Pharmingen), PE-conjugated anti-CD11b (BEAR-1, Beckman Coulter), PerCP-conjugated anti-CD20 (clone B9E9, Immunotech), PE-conjugated anti CD8 (clone RPA-T8, Becton Dickinson) and PerCP-conjugated antiCD4 (clone L200, BD Pharmingen). Cells were washed twice and fixed in CellFix solution (Becton Dickinson, Erembodegem, Belgium) for 3 days before analysis on a Becton Dickinson FACS apparatus with CellQuest Software (Becton Dickinson). eGFP fluorescence was detected in the isothiocyanate (FITC) channel. Negative controls from normal macaques were run with every experimental sample and were used to establish gates for eGFP quantification.

### Polymerase chain reaction (PCR) assays

Cellular DNA was extracted from peripheral blood mononuclear cell (PBMC) samples, using the High Pure PCR Template Preparation Kit according to the manufacturer's instructions (Roche Mannheim, Germany). DNA was quantified by measuring optical density (Spectra Max 190; Molecular Devices, California, USA). The eGFP sequence was analyzed by quantitative real-time PCR on 250 ng of DNA run on an iCycler real-time thermocycler (Bio-Rad, California, USA). Primers were as follows: forward primer, 5'ACGACGGCAACTACAAGACC3'; reverse primer, 5'GCCATGATATAGACGTTGTGG3'. Amplification was performed in a final volume of 50 μl, with IQ™ SYBR^®^Green Supermix (Bio-Rad, California, USA), in accordance with the manufacturer's instructions. Amplification was carried out over 40 cycles of denaturation at 95°C, annealing at 59°C and elongation at 72°C. Standard curves for the eGFP sequence were generated by serial 10-fold dilutions of duplicate samples of the eGFP plasmid in DNA from untransduced PBMC, with 250 ng of total DNA in each sample. Samples from animals were run in duplicate, and the values reported correspond to the means for replicate wells.

### Statistical analysis

Paired and unpaired comparisons were performed using non parametric Kruskal Wallis, Wilcoxon rank and Mann & Whitney tests, respectively, both of which can be used for the analysis of small samples when normal distribution is uncertain or not confirmed. Tests were performed using StatView 5.01 sofware (Abacus Concepts, Berkeley, CA).

## Results

### Efficient transduction of cynomolgus macaque CD34^+ ^bone marrow cells

**Figure 2:**
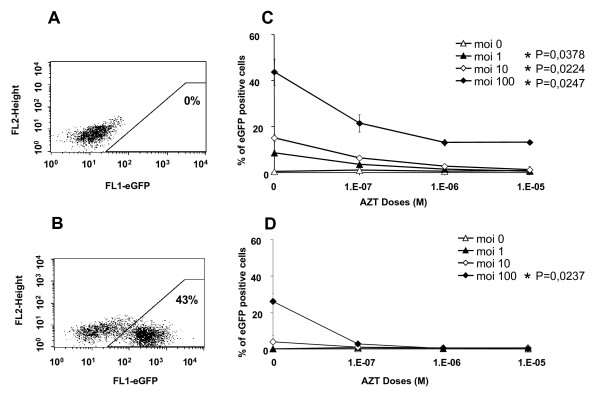
Efficiency of transduction of cynomologus macaque primitive hematopoietic cells with SIV-based lentiviral vectors. A: Non transduced cells were used as a control for each animal. B: Transduction of bone marrow progenitor cells with an SIV-based vector. CD34^+ ^cells were cultured in the presence of cytokines (see materials and methods) and exposed to vector particles at an MOI of 100 for 24 hours before FACS analysis for eGFP production. C: CD34^+ ^cells were cultured overnight in a proliferation medium supplemented with various concentrations of AZT (100 nM, 1 mM, 10 mM). Cells were then washed twice and transduced with various multiplicities of infection (MOI) of the lentiviral vector (0, 1, 10, 100). After 24 hours of coculture with lentiviral vector, some of the CD34^+ ^cells were used to evaluate the rate of transduction of undifferentiated CD34^+ ^cells (C); * indicate statistically significant differences (Kruskal Wallis test) between cultures with and without AZT treatment for MOI = 1 (p = 0,0378), MOI = 10 (p = 0,0224) and MOI = 100 (p = 0,0247). Some of the cells were cultured for 14 days, to allow the myeloid differentiation of CFC. Cells were then resuspended, washed and fixed for three days. They were analyzed by flow cytometry, to evaluate the percentage of eGFP-positive cells and determine the rate of transduction (D); * indicates a statistically significant difference (p = 0,0237(Kruskal Wallis test)) between cultures with and without AZT treatment for MOI = 100. The results shown are the mean values for the three monkeys, each studied in triplicate.

We first assessed, *in vitro*, the efficiency with which a SIVmac251-derived vector transduced CD34^+ ^hematopoietic cells from macaque bone marrow (BM). We harvested BM cells from the iliac crests of 12 different animals. CD34^+ ^cell preparations with a purity of 97% ± 1% were obtained by immunomagnetic purification. The CD34^+ ^cells were then transduced by coculture for 24 h with the lentiviral vector (MOI = 100) in medium supplemented with SCF, Flt3-L, IL-3 and IL-6. The vector used (pRMES8) was derived from SIVmac251 and contains the eGFP reporter gene under control of the phosphoglycerate kinase (pGK) promoter (Figure 1). Transduction efficiency (Figure 2A and 2B), as evaluated by flow cytometry analysis of eGFP expression at 24 h, was 41% ± 9% on average (n = 12). After 24 hours of culture with the lentiviral vector, some of the purified CD34^+ ^cells were cultured for 14 days in semi-solid medium containing SCF, GM-CSF, IL-3 and EPO to allow the myeloid differentiation of colony-forming cells (CFC), whereas some cells were cocultured for 35 days on a layer of murine fibroblasts of the M2-10B4 cell line and were then cultured for 14 days on semi-solid medium containing SCF, GM-CSF, IL-3 and EPO, for the identification of long-term culture-initiating cells (LTC-IC). Transduction had no effect on the clonogenic capacity of CD34^+ ^cells: the mean number of colonies was 41 ± 10 for non transduced cells and 44 ± 12 for pRMES8-transduced cells (12 animals tested, P = 0.60 (Mann & Whitney test)). Similar results were obtained for LTC-IC, with 19 ± 3 colonies obtained for non transduced cells and 19 ± 3 for transduced cells (n = 12; P = 0.79 (Mann & Whitney test)). Transduction rates did not differ significantly between CFC and LTC-IC (P = 0.4884 (Wilcoxon test), n = 12), with 18% ± 7% and 19% ± 7% of colonies, respectively, eGFP-positive. However, in both cases, the percentage of eGFP-positive cells was significantly lower than that observed 24 hours after transduction (P < 0.0001 (Wilcoxon test)). This apparent discrepancy between analyses carried out at 24 h and analyses on CFC or LTC-IC may be due to the eGFP protein present in viral particles and incorporated into the cell cytoplasm during the coculture period. The proportion of cells producing eGFP shortly after transduction was reduced by 25% ± 15% (Figure 2C) if 10^-6 ^M AZT was added to cocultures of CD34^+ ^BM cells and lentiviral vector (MOI = 100). Untreated CFC cultures gave percentages of eGFP-producing cells similar to those observed before differentiation (26% ± 5%) (Figure 2D). No fluorescence was detected after myeloid differentiation of the AZT-treated CFC (n = 3), confirming that eGFP detection resulted from the production of this protein from integrated vector.

**Figure 3:**
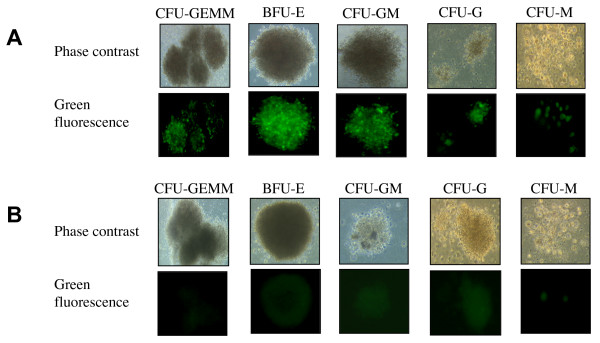
Fluorescence microscopy after myeloid differentiation of CFC (×100). Freshly isolated CD34^+ ^cells were transduced or not with the lentiviral vector (24 hours of culture with lentiviral vector at MOI = 100). Cells were then cultured for 14 days in the presence of cytokines, to allow myeloid differentiation of transduced (A) and not transduced (B) CD34+ cells. Abbreviations: CFU-GEMM, Colony-Forming Unit-Granulocytes, Erythroid, Macrophage, Megakaryocyte; BFU-E, Burst-Forming Unit-Erythroid; CFU-GM, Colony-Forming Unit-Granulocytes, Macrophage; CFU-G, Colony-Forming Unit-Granulocytes; CFU-M, Colony-Forming Unit-Macrophage.

Mosaicism was observed in eGFP gene expression in several colonies (Figure 3). Indeed, eGFP was detected in 56% ± 4% of colonies, whereas only 26% ± 5% of individual cells were eGFP-positive. These results suggest that, on average, only 47% of cells from a single colony contained the SIV vector.

### Transplantation of autologous BM CD34^+ ^cells transduced by SIV-based vector into cynomolgus macaques

We explored the capacity of autologous CD34^+^BM cells transduced *ex vivo *with a lentiviral vector to engraft efficiently into macaques after total body irradiation (TBI) with a gamma source at the sublethal dose of 6 Gy. Three groups of 4 animals were used: 1) In Group 1, macaque CD34^+ ^BM cells (96% ± 1% pure on average) were obtained from the two humeri before gamma irradiation (Table 1). These cells were cocultured, as described above, with pGASE, which is an improved version of pRMES8. Indeed, a mean transduction efficiency of 72% ± 4% was obtained (n = 4) at 24 hours and 37% ± 10% of CFC produced eGFP. Two days after gamma irradiation, 1.4 × 10^6 ^to 2.9 × 10^6 ^CD34^+ ^cells per kg were injected into both humeri of macaques (Table 1); 2) Group 2 included irradiated (6 Gy) macaques that did not undergo cell transplantation: 3) Group 3 included 4 non irradiated animals, which were used as controls, with a similar bleeding frequency.

### Reconstitution of hematopoietic cells *in vivo*

**Figure 4:**
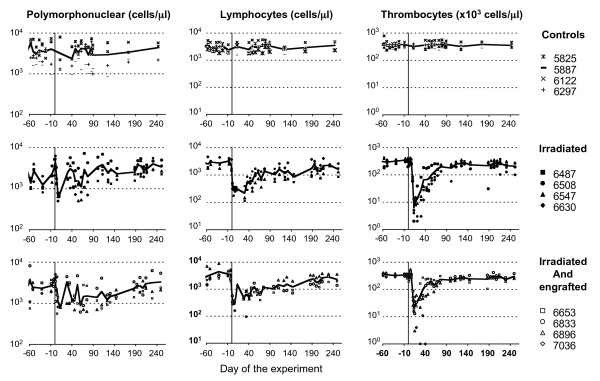
Effect of irradiation and transplantation on polymorphonuclear cell, lymphocyte and thrombocyte counts. All animals were followed during the weeks preceding the study, and for more than 240 days after the irradiation. We carried out hematological analysis including blood cell counts with an automated hemocytometer (Coulter Corporation, Miami, USA).

Following total-body irradiation with 6 Gy, transfusion and an antibiotic regimen were required to ensure that all the animals survived. However, one animal from group 1 (7036) died on day 40 due to profound pancytopenia (Figure 4). This macaque received the smallest number of autologous and transduced CD34^+ ^BM cells. All other animals from groups 1 and 2 were studied from days -1 to 471 after gamma irradiation. Controls were followed over the same period.

Radiation rapidly induced severe anemia in all animals (data not shown). A significant decrease in the number of polymorphonuclear cells in the periphery was observed, starting on day 1 after irradiation (Figure 4). No significant difference was observed between the animals of groups 1 and 2 in terms of the minimum number of cells (821 ± 226 cells/μl for group 1 and 658 ± 107 cells/μl for group 2, P = 0.3768 (Mann & Whitney test)) or the time at which that minimum occurred (6 ± 5 days for group 1 and 7 for group 2, P = 0.4795 (Mann & Whitney test)). Lymphocyte counts also decreased in all macaques by day 1 after gamma irradiation (Figure 4), falling to a minimum of 220 ± 107 lymphocytes/μl on day 18 ± 12 in group 2 and of 347 ± 62/μl on day 11 ± 12 in transplanted animals (group 1). Animals undergoing transplantation tended to display less severe lymphopenia, but no statistical difference was observed between the two groups of irradiated animals in terms of the day on which minimum lymphocyte count was reached (P = 0.1939 (Mann & Whitney test)) or the level of that minimum (P = 0.3805 (Mann & Whitney test)). A significant decrease in platelet counts, beginning by day 10 (Figure 4), was observed in all irradiated animals. Thrombocytopenia (platelet count < 20,000/μl) was characterized in non transplanted animals by a minimum value of 3.75 ± 2.49 × 10^3 ^platelets/μl on day 18 ± 3. Thrombocytopenia tended to be less severe in transplanted animals, but this difference was not significant for the minimum number of platelets (10.33 ± 5.25 × 10^3 ^platelets/μl; P = 0.1124 (Mann & Whitney test)) or for the day on which that minimum occurred (14.33 ± 0.94; P = 0.3123 (Mann & Whitney test)). This thrombocytopenia required one transfusion in all animals (other than animal 7036, which needed two transfusions) of both groups. However, platelet reconstitution seemed to be correlated with the dose of CD34^+ ^cells infused, the speed of reconstitution increasing with the number of CD34^+ ^cells injected (macaque 6653).

### Reconstitution of bone marrow clonogenic activity

**Figure 5:**
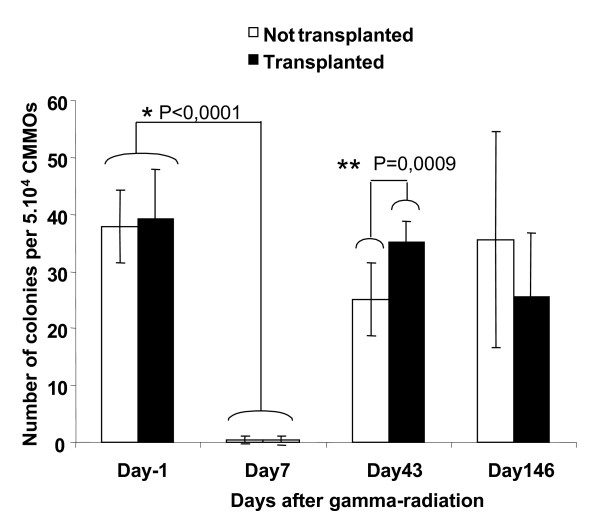
Recovery of bone marrow clonogenic activity. Bone marrow-derived colony-forming units following sublethal irradiation of cynomolgus monkeys transplanted (black bars) or not transplanted with CD34^+ ^cells (open bars). Mean ± SD of CFC number (triplicate). The results of statistical test are indicates; * indicates a statistically significant difference (p < 0,0001 (Wilcoxon test)) between day 0 and day 7 for the both group; ** indicates a statistically significant difference (p = 0,0009 (Mann & Whitney test)) at day 43 between animals undergoing transplantation and those that did not undergo transplantation.

We determined the effects of CD34^+ ^bone marrow cell transplantation following gamma irradiation on the *ex vivo *proliferation and differentiation of hematopoietic progenitors. Before gamma irradiation, a mean of 40 ± 9 and 38 ± 6 colonies was observed for groups 1 and 2, respectively (Figure 5). Colony number decreased significantly (P < 0.0001 (Wilcoxon test)) by day 7 in all animals. In both groups, clonogenic activity was detected by day 43 after gamma irradiation with reconstitution significantly better in the animals undergoing transplantation than in those that did not undergo transplantation (P = 0.0009 (Mann & Whitney test)).

### Presence of eGFP-positive cells in bone marrow and peripheral blood

Cells with integrated SIV-vector DNA were detected by PCR (Table 2) as early as day 3 after transplantation, in at least two animals (6653 and 6833). These two animals had received the largest numbers of transduced CD34^+ ^bone marrow cells. Monkey 7036, which died within 40 days of gamma irradiation had very few transduced cells in the bone marrow and SIV-DNA was not detected in peripheral blood cells. In the three remaining animals, vector DNA was detected in peripheral blood cells (up to 500 copies per million cells) and in the bone marrow (up to 6250 copies per million cells) more than one year after transplantation (day 471).

**Table 2:** Number of DNA copies per million mononuclear cells in peripheral blood (PB) and bone marrow (BM)

	Monkey							
	6653		6833		6896		7036	
Days post transplantation	PB	BM	PB	BM	PB	BM	PB	BM

-3	0	0	0	0	0	0	0	0
3	500	ND	250	ND	0	ND	0	15
5	250	500	ND	250	ND	ND	0	0
108	250	ND	250	ND	1250	ND	*	*
121	750	ND	250	ND	250	ND	*	*
128	250	ND	250	ND	250	ND	*	*
142	250	ND	250	ND	1750	3250	*	*
471	ND	250	250	250	500	6250	*	*

ND: not determined

*: 7036 died on day 40

**Figure 6:**
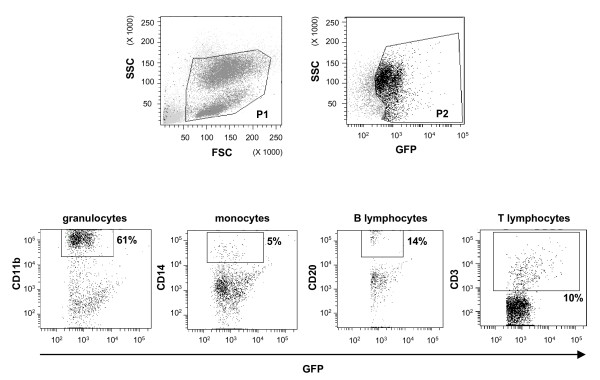
Flow cytometry analysis of hematopoiesis reconstitution. Animal transplanted with autologous CD34^+ ^bone marrow cells transduced with an SIV-based vector. eGFP-positive cells present in P1 and P2 were analyzed by immuno-staining to identify the subpopulations of eGFP-positive cells in peripheral blood. CD20-PerCP-Cy5, CD14-PE, CD11b-APC and CD3-APC staining were used to identify the B-lymphocyte, monocyte, granulocyte and T-lymphocyte subpopulations.

Flow cytometry analysis demonstrated the presence of eGFP-producing cells among peripheral blood mononuclear cells in myeloid and lymphoid lineges of monkey 6896 (Figure 6). Peripheral blood cells were sorted on the basis of eGFP production, with the aim of characterizing the phenotype of populations of cells expressing the transgene in more detail. We found that 61% of eGFP-positive cells were CD11b-positive,5% of these cells appeared to be CD14+ monocytes, 14% were CD20^+ ^B cells and 10% were CD3+ T cells, 23% of which expressed CD8 and 77% expressed CD4 (data not shown).

## Discussion

The aim of this work was to study reconstitution of the myeloid and lymphoid compartments after the autologous transplantation of genetically modified CD34^+ ^bone marrow cells into cynomolgus macaques previously subjected to gamma irradiation.

We first assessed, *in vitro*, the efficiency with which a SIVmac251-derived vector transduced macaque CD34^+ ^hematopoietic bone marrow cells. These vectors are similar to those derived from HIV. However, SIV-derived vectors clearly outperform HIV-derived vectors in simian cells. In fact, HIV-1 fails to replicate in simian cells because of an early postentry block [43,44], and Kootstra *et., al *showed that the viral determinant involved in postentry restriction of HIV-1 replication in simian cells is located at or near the cyclophilin A (CyPA) binding region of the capside protein [45]. The hydrophobic pocket of cyclophilin A (CypA) makes direct contact with an exposed, proline-rich loop on HIV-1 capsid (CA) and renders reverse transcription complexes resistant to an antiviral activity in human cells. A CypA fusion with TRIM5 (a member of the tripartite motif family) that is unique to New World owl monkeys also targets HIV-1 CA, but this interaction potently inhibits infection. A similar block to HIV-1 infection in Old World monkeys is attributable to the α isoform of the TRIM5 orthologue in these species and using RNA interference techniques, Berthoux *et., al *demonstrated that CypA inhibits HIV-1 replication in these cells because it is required for CA recognition by TRIM5α [46]. SIV vectors can also efficiently transduce human cells[33,47], and may therefore prove a useful alternative to HIV-1-based vectors, at least in the early phase of preclinical testing of lentivirus vectors. We found that the proportion of eGFP-positive cells obtained before myeloid differentiation (mean value of 30%) was similar to that obtained with CD34^+ ^cells from human donors transduced with lentiviral [48-51], retroviral [52-54], AAV[55], or adenovirus/AAV-derived [56] vectors. However, it is possible to increase the transduction rate, such that 90% transduced human CD34^+ ^cells are obtained from cord blood, 80% from bone marrow and 75% from G-CSF mobilized peripheral blood [57]. We analyzed transduction in two types of assay, based on committed (CFC) and primitive (LTC-IC) hematopoietic progenitors, as analyses of the transduction of committed progenitors only bear little relation to the transduction efficiency for stem cells and less differentiated cells in the long term. After myeloid differentiation, eGFP^+ ^cells were detected, in similar proportions, in CFC on day 15 and LTC-IC on day 50 after transduction, indicating that the vector was able to transduce progenitor cells and most immature hematopoietic cells with a similar efficiency. Similar results have been reported for stimulated human CFC and LTC-IC, which were found to be transduced with similar efficiency by a lentiviral vector based on HIV-1[58]. In this previous study, significant resistance to lentiviral transduction was reported in unstimulated primitive human cells. These results may explain why, in our study, the use of cytokines during transduction made possible the genetic modification of LTC-IC, which are quiescent. Cytokine treatment may have led to these cells entering the cell cycle, facilitating transduction. This result confirms the greater efficiency of lentiviral vectors than of retroviral vectors for the transduction of CD34^+ ^cells. Nevertheless, in our study, only half as many eGFP-positive cells were obtained after differentiation as were obtained from undifferentiated CD34^+ ^cells. Similar observations have been made with MLV-transduced progenitor cells from human donors[59]. We demonstrate here that these differences may be accounted for by the pseudotransduction detected at 24 h of incubation with the vector, confirming the results reported with CD34^+ ^cells in studies using VSVg-pseudotyped MLV-derived[60] or lentivirus-derived vectors[51]. It has been suggested that pseudotransduction may result from VSVg-pseudotyping due to membrane fusion efficiency being higher than the rate of integration of the transgene[61]. Nevertheless, most lentiviral vectors have been generated with VSV-G, as this glycoprotein makes it easy to recover and concentrate the pseudotyped vectors [62].

We also showed that eGFP was produced in all colony subtypes. Clusters of eGFP production were observed on fluorescence microscopy, indicating that not all the cells of a given positive colony – theoretically derived from a single cell – produced eGFP. This result is consistent with those of Mikkola *et al. *concerning murine HSC transduction by a VSVg-pseudotyped lentiviral vector, in which a mismatch was reported between the transduction rate of cells (almost 25%) and the transduction rate of myeloid colonies (almost 60%). These authors highlighted the occurrence of mosaicism in GFP gene expression in colonies obtained following the myeloid differentiation of CD34^+ ^cells[63], possibly due to a delay in the integration of the transgene during differentiation, resulting in the formation of clusters of GFP-positive cells within a single myeloid colony.

In our *in *vivo study, autologous HSC were injected into the bone marrow, whereas intravenous injection is currently the most frequently used transplantation method. We aimed to increase seeding efficiency and homing, as only a limited number of stem cells were theoretically available. However, 2.5 × 10^6 ^to 5.0 × 10^6 ^CD34^+ ^cells is generally sufficient to ensure engraftment, and we found that less than 2.0 × 10^6 ^cells were sufficient for long-term reconstitution in macaques. As predicted[64,65], total-body gamma irradiation leads to a drastic decrease in the number of hematopoietic progenitors, preventing the development of mature cells [66]. Despite the occurrence of severe pancytopenia, a positive correlation has been found between the number of CD34^+ ^cells infused and time required for immune reconstitution [42,67,68]. However, hematopoietic recovery may take longer if fewer than 2.0 × 10^6 ^CD34^+ ^cells/kg are infused. This notion is consistent with our observation that CD34^+ ^cell transplantation decreases both the severity and duration of irradiation-induced cytopenia. Clonogenic activity also reappeared more strongly in transplanted animals. We also showed that the animals recovered B cells, T cells, monocytes and granulocytes. Nevertheless, the functional activity of these cells requires confirmation, particularly for lymphocytes. However, although we observed long-term reconstitution with lentiviral vector-transduced cells of different lineages, its proportion remained below 1%. Hanawa *et al*., provided the first evidence that SIV-based vectors can successfully transduce rhesus macaque repopulating hematopoietic stem cells, with an average of 16% of peripheral blood leukocytes containing the SIV vector genome. However, this study was carried out with HSC from mobilized peripheral blood cells, making it possible to obtain larger numbers of HSC than can be harvested from bone marrow. Nevertheless theoretically, these cells contained more progenitors that were already committed and fewer pluripotent stem cells capable of long-term reconstitution than medullary HSC[69]. The small numbers of eGFP-producing cells observed in our study may be due to an anti-eGFP immune response. Some reports have suggested that such reactions do not generally occur after irradiation[70], but two reports described the induction of cytotoxic T-lymphocyte responses to enhanced green (GFP) or yellow (YFP) fluorescent proteins after myeloablative conditioning. One of these reports concerned baboons that had received primitive hematopoietic cells transduced with HIV-1-based lentiviral vectors[71] and the other concerned rhesus macaques that had received CD34^+ ^stem cells transduced with a retroviral vector[72].

Lentiviruses, like retroviruses, can be used to integrate transgenes into the host genome. Two severe adverse events occurred in two patients in the SCID-X1 gene therapy trial 30 to 34 months after injection of the autologous CD34^+ ^cells corrected using a retroviral vector. In these patients, an uncontrolled clonal T lymphoproliferative syndrome, similar to acute lymphoblastic leukemia, was observed [73,74]. This study highlights the risk of insertional mutagenesis restricted to retroviral and lentiviral gene transfer. In the future, additional safety measures could be considered, such as the use of self-inactivating LTRs (as in our study) to reduce enhancer activity, the addition of insulators to reduce the risk further, and the insertion of a second transgene encoding a "suicide" product, such as herpes thymidine kinase, making it possible to kill the transduced cells with ganciclovir. Unlike studies in mice, in which the follow-up period is necessarily limited, studies in large animals, with a longer life span, are compatible with more extensive follow-up. The development of linear amplification-mediated PCR (LAM-PCR), a sensitive and robust approach to molecular clonal analysis, has made it possible to identify and analyze the contribution of individual transduced clones to hematopoiesis. Clonal analysis may provide information about the dominance of transduced clones, potentially predicting possible progression or the propensity to develop clonal hematopoiesis and leukemia. Moreover, replication-competent retrovirus (RCRs), recombinant retrovirus and interaction with endogenous retroviruses should also be investigated, when evaluating the biosafety of retrovirus and lentivirus.

## Conclusion

The results reported here provide the first evidence that gene transfer into medullary hematopoietic stem cells and long-term expression of the transgene are possible, using an SIV-based lentiviral vector in non human primates, which provide the best clinical models for *in vivo *evaluation of the feasibility and safety of gene therapy strategies.

## Competing interests

The authors never received reimbursements, fees, funding, or salary from an organization that may in any way gain or lose financially from the publication of this paper. The authors never have any stocks or shares in an organization that may in any way gain or lose financially from the publication of this paper. The authors have no competing interests to declare in relation to this paper.

## Authors' contributions

SD was the main contributor to this paper. This work is part of her PhD project. She carried out transduction of CD34^+ ^cells, transplantation of animals, PCR for identification of cells expressing the transgene *in vivo*, flow cytometry analysis, WG Have improved assays for transduction of macaque bone marrow CD34^+ ^cells with SIV derived vector, DN constructed and produced the SIV derived vector, SP technical assistance to cell sorting, MLD technical assistance to transplantation, BD technical assistance to cell culture, flow cytometry and irradiadion of NHP, GA technical assistance to molecular biology, TA technical assistance to flow cytometry and cell sorting, JLL irradiation of animals and dosimetry, FLC supervises vector design and production, RLG supervisor of SD.
